# Depth-driven patterns in lytic viral diversity, auxiliary metabolic gene content, and productivity in offshore oligotrophic waters

**DOI:** 10.3389/fmicb.2023.1271535

**Published:** 2023-11-02

**Authors:** Anastasia Tsiola, Grégoire Michoud, Daniele Daffonchio, Stilianos Fodelianakis, Antonia Giannakourou, Dimitris Malliarakis, Alexandra Pavlidou, Elli Pitta, Stella Psarra, Ioulia Santi, Christina Zeri, Paraskevi Pitta

**Affiliations:** ^1^Institute of Oceanography, Hellenic Centre for Marine Research (HCMR), Heraklion Crete, Greece; ^2^Biological and Environmental Sciences and Engineering Division (BESE), Red Sea Research Center, King Abdullah University of Science and Technology (KAUST), Thuwal, Saudi Arabia; ^3^Institute of Oceanography, Hellenic Centre for Marine Research (HCMR), Anavyssos, Attiki, Greece; ^4^Department of Biology, University of Crete, Heraklion Crete, Greece

**Keywords:** metagenomics, oligotrophy, viral auxiliary metabolic genes, lytic production, Eastern Mediterranean Sea

## Abstract

**Introduction:**

Marine viruses regulate microbial population dynamics and biogeochemical cycling in the oceans. The ability of viruses to manipulate hosts’ metabolism through the expression of viral auxiliary metabolic genes (AMGs) was recently highlighted, having important implications in energy production and flow in various aquatic environments. Up to now, the presence and diversity of viral AMGs is studied using -omics data, and rarely using quantitative measures of viral activity alongside.

**Methods:**

In the present study, four depth layers (5, 50, 75, and 1,000 m) with discrete hydrographic features were sampled in the Eastern Mediterranean Sea; we studied lytic viral community composition and AMG content through metagenomics, and lytic production rates through the viral reduction approach in the ultra-oligotrophic Levantine basin where knowledge regarding viral actions is rather limited.

**Results and Discussion:**

Our results demonstrate depth-dependent patterns in viral diversity and AMG content, related to differences in temperature, nutrients availability, and host bacterial productivity and abundance. Although lytic viral production rates were similar along the water column, the virus-to-bacteria ratio was higher and the particular set of AMGs was more diverse in the bathypelagic (1,000 m) than the shallow epipelagic (5, 50, and 75 m) layers, revealing that the quantitative effect of viruses on their hosts may be the same along the water column through the intervention of different AMGs. In the resource- and energy-limited bathypelagic waters of the Eastern Mediterranean, the detected AMGs could divert hosts’ metabolism toward energy production, through a boost in gluconeogenesis, fatty-acid and glycan biosynthesis and metabolism, and sulfur relay. Near the deep-chlorophyll maximum depth, an exceptionally high percentage of AMGs related to photosynthesis was noticed. Taken together our findings suggest that the roles of viruses in the deep sea might be even more important than previously thought as they seem to orchestrate energy acquisition and microbial community dynamics, and thus, biogeochemical turnover in the oceans.

## 1. Introduction

Viruses are the most abundant biological entities in the oceans and have a large impact on biogeochemical processes. Indeed viruses modulate the cycling of nutrients, since organic matter is released from the lysed cells and returns to prokaryotes in readily-available dissolved form rather than being transferred to higher trophic levels (Suttle, 2007). Viral activity also results in the formation of gelatinous, aggregated particles from the products of lysis and through this process, organic material is diverted from surface layers to deeper ones (Zimmerman et al., 2020).

But even without lysing their hosts, viruses may reprogram hosts’ metabolism and thus alter nutrients flow, through the expression of viral Auxiliary Metabolic Genes (AMGs). AMGs are expressed by viral genomes when the host is infected and still intact; their expression causes a “shift” in hosts’ metabolism toward viral replication, for instance through enhancement of microbial nutrient uptake under low nutrients availability. This pattern was particularly studied under P limited conditions (Zeng and Chisholm, 2012; Kelly et al., 2013; Lin et al., 2016) and resulted in boosted host survival and viral replication too. Expression of AMGs has been described at the transcriptional level mostly in cultures (Lin et al., 2016; Bachy et al., 2018; Howard-Varona et al., 2020) and rarely in natural communities (Sieradzki et al., 2019; Heyerhoff et al., 2022) but also at the genome level in various ecosystems including the open and coastal ocean (Crummett et al., 2016; Luo et al., 2020; Tsiola et al., 2020; Jian et al., 2021). Special focus has been given on photosynthesis and carbon-metabolism related AMGs (Thompson et al., 2011) as well as on deciphering the wide variability of phage-host interactions that lead to totally different host metabolisms (Howard-Varona et al., 2018, 2020).

High-throughput exploration of viral populations is nowadays predominant (Guo et al., 2021; Nayfach et al., 2021; Pons et al., 2021; Gregory et al., 2022; Roux et al., 2023) in comparison to earlier studies that mostly applied flow cytometry (Marie et al., 1999) despite the fact that the usual nucleic acid stains may have underestimated viral counts. Viral community structure and diversity is now known across spatial and temporal scales (Mizuno et al., 2013; Hurwitz et al., 2015; Paez-Espino et al., 2016; Coutinho et al., 2019; Gregory et al., 2019; Luo et al., 2020; Tsiola et al., 2020; Wu et al., 2020). Global expeditions revealed that patterns of diversity are linked to temperature as well as oxygen and nutrients concentration and *Prochlorococcus* abundance (Brum et al., 2015; Coutinho et al., 2017; Gregory et al., 2019). Similarly to diversity, selection of AMGs was also found to be driven by environmental variables at the global and smaller scales, mainly by temperature and depth (Williamson et al., 2008). Temperature in specific seems to control not only diversity patterns (Steward et al., 2000; Hurwitz et al., 2013, 2015) but also viral abundances and cytometric characteristics (Winter et al., 2012).

Next, to the so called “qualitative” implications, the most straightforward implications of viruses in the oceans are quantitative; viruses cause mortality, i.e., removal of standing stock and release of carbon that may reach approx. 10 billion tons day^−1^ in the oceans (Suttle, 2007; Brussaard et al., 2008). Quantitative data, such as the frequency of lytically-infected and lysogenic cells, mostly originate by applying the viral reduction approach (Wilhelm et al., 2002; Winget et al., 2005). The dynamics of infections seem to be highly related to temperature (Mojica and Brussaard, 2014) and depth (Brum, 2005) but also to hosts’ abundance and productivity (Brum et al., 2016) and organic matter and energy availability (Lara et al., 2017).

To link quantitative and qualitative implications, relevant data needs to be worked together. We hereby describe viral abundances and lysis rates, viral community structure, and AMG content in the lytic fraction (<0.2 μm) in relation to the environmental setting of a largely unexplored basin in the Eastern Mediterranean Sea (EMS). The EMS is a semi-enclosed, concentration-type basin where evaporation largely exceeds precipitation and river run-off. It has unique circulation patterns (Velaoras et al., 2019) and physicochemical features, including high bottom-water temperatures, salinity and transparency, extreme oligotrophy and limited microbial plankton growth associated mainly to low phosphate and inorganic nitrogen sources (Azov, 1991; Krom et al., 2004). We sampled the Levantine Basin, the easternmost part of the EMS, at 5, 50, 75, and 1,000 m depths in order to investigate water masses with discrete temperature, density, and levels of oxygen, nutrients and chlorophyll; these factors that are among the most frequent controlling the microbial communities, including the viral component (Techtmann et al., 2015). According to Velaoras et al. (2019) who performed the hydrographic study in this cruise, distinct water masses are encountered in the studied area from the surface to the seafloor. Down to 75 m, Atlantic Water and Levantine Surface Water masses are rather warm, saline and saturated in oxygen, with no major differences in current distributions across the vertical scale of this depth layer. Levantine Surface Water masses are mostly prominent in the easternmost side of the sampling area (LV13 and LV18 sampling stations, see section 2.1), while the westernmost side (LV3 and LV10 sampling stations, see section 2.1) is additionally affected by a branch of Mid-Mediterranean Jet that carries low-salinity Atlantic Water from the Ionian to the Levantine Sea. Deeper than 75 m, the intermediate layer extends down to ~165 m, and further below, three water masses can be identified: the Transitional Mediterranean Water between ~600 and 1,200 m, the old Cretan Deep Water between ~1,400 and 2,500 m, and the Eastern Mediterranean Deep Water below ~3,000 m. As part of a multidisciplinary sampling expedition with pre-defined sampling depths, we chose to collect water from 5 m (representing surface water), 75 m [representing the deep euphotic layer, approaching the deep-chlorophyll maximum (Livanou et al., 2019)] and 50 m (representing an additional depth within the mixed layer and the thermocline, with nearly equal primary productivity levels as the deep-chlorophyll maximum, not published data). All surface/subsurface depths were chosen so they remain within the mixed layer (during the sampling time, stratification of the water column was just starting) and within the thermocline. Further, we chose to collect water from 1,000 m [representing a depth layer dominated by Transitional Mediterranean Water masses, rich in nutrients and of low salinity and oxygen (Velaoras et al., 2019)]. We coupled traditional and modern ecological and genomic approaches to shed light on ecosystem processes that are affected by viral actions. We expected to find discrete viral populations in terms of activity and genomic signature in the coldest waters in the bathypelagic zone, dependent on temperature and energy. Sampling constraints and high analytical costs did not allow us to study a higher number of replicated sampling stations and seasons. Thus, we wish to point out that the presented findings should be generalized to other ecosystems/seasons with caution, and that further testing is needed to confirm our hypotheses. According to previous findings in the same project that revealed depth-specific eukaryotic community patterns (Santi et al., 2020) and other recent similar surveys (Coutinho et al., 2019, 2023), we hypothesized that a wider repertoire of viral AMGs characterize the cold bathypelagic waters, available to aid energy acquisition under resource-depleted conditions.

## 2. Materials and methods

### 2.1. Sampling

Sampling was carried out on board the R/V Aegaeo in April 2016, at the onset of seasonal thermal stratification. Seawater was collected between 09:00 and 12:00 am by Rosette-Niskin deployment from 4 stations in the Western Levantine basin (Eastern Mediterranean Sea), encoded as LV3 (35.0333oN, 23.4667oE), LV10 (34.6667oN, 24.3667oE), LV13 (34.2500oN, 25.4833oE), and LV18 (34.4333oN, 26.3833oE). Four depth layers were sampled in each station: 5, 50, 75, and 1,000 m. The collected volume was transferred from the Niskin bottles into acid-cleaned and deionized water-rinsed low-density polyethylene containers, and further processed for the various analyses.

### 2.2. Assessment of physical and chemical variables

Hydrographic profiles of salinity (*S*), temperature (T) and density (D) were obtained using a Seabird SBE 9plus CTD underwater unit connected to a Seabird SBE 11 deck unit (Velaoras et al., 2019). The methodologies for the determination of dissolved oxygen (DO), nitrate (NO_3_^−^), nitrite (NO_2_^−^), silicate (SiO_4_), ammonium (NH_4_), phosphate (PO_4_^3−^), dissolved organic carbon (DOC), phosphorus (DOP) and nitrogen (DON), total nitrogen (TN) and phosphorus (TP) and chlorophyll a (Chl) concentrations are presented in the Supplementary material.

### 2.3. Assessment of viral and bacterial abundances

The abundance of virus-like particles (VLP) was determined based on the protocol of Brussaard (2004) and that of heterotrophic and autotrophic bacteria based on Marie et al. (1997) from glutaraldehyde-fixed samples (0.5% final concentration). Samples were stained with SYBR™ Green I nucleic acid stain (ThermoFisher Scientific) at 5 × 10^−5^ and 4 × 10^−4^ final dilution of the stock solution for VLP and heterotrophic bacteria, respectively, and then incubated for approx. 15 min at 80°C and for approx. 30 min in the dark for VLP and heterotrophic bacteria, respectively. Autotrophic bacteria were distinguished based on their auto-fluorescence signals. Yellow-green latex beads of 1 μm nominal size (Polysciences) were added and used as an internal standard of fluorescence. A FACSCalibur™ instrument (Becton Dickinson) was used at conventional air pressure, with an air-cooled laser at 488 nm and standard filter setup. Data were processed with the CellQuest™ Pro software (Becton Dickinson).

### 2.4. Assessment of viral and bacterial production rates

Viral production rates were estimated following the viral reduction approach (Winter et al., 2004; Winget et al., 2005). Two L of seawater were pre-filtered through 0.8 μm pore size polycarbonate membranes to exclude large particles and grazers. The 0.8 μm-filtrate was concentrated using a tangential flow filtration system (Sartorius, VF20P7, 0.2 μm MWCO). The 0.22-μm filtrate was back-flushed with the use of the reverse flow mode of a peristaltic pump (Masterflex, EW-07523-80) in order to produce an ultra-concentrated-in-bacteria seawater volume (100 mL). The remaining 0.22-μm filtrate was further filtered with a spiral-wound ultra-filtration cartridge (Sartorius, VF20P2, 30.000 MWCO) so to produce a virus-free ultra-filtered volume (200 mL). Bacterial-concentrated and virus-free water masses were mixed gently, and then equally distributed into 50-mL centrifuge tubes (triplicate incubations). Tubes were incubated at *in situ* temperature (as determined by CTD) and in the dark for 24 h. Every 0, 1, 3, 6, 12, and 24 h during the incubation, samples were fixed for the determination of viral and bacterial abundances as described above. Lytic viral production (lytic VP, expressed in virus-like particles mL^−1^ h^−1^) was estimated from the slopes of the relationships between net increases in viral abundance over the respective time period of the net increase.


$$VP=\left[\frac{\mathrm{VLPmax}-\mathrm{VLPmin}}{\mathrm{tmax}-\mathrm{tmin}}\right]$$

Lytic VP was further corrected for the bacterial losses due to the filtration; the recovery percentage was determined by dividing the mean bacterial abundance at the onset of the incubations (B0) by the *in situ* bacterial abundance (B original), and this factor was multiplied to the lytic VP measurement.


$$\mathrm{Lytic}\;VP=VP\;x\;\left[\frac{\mathrm{Boriginal}}{B0}\right]$$

The methodology for the assessment of heterotrophic bacterial production is presented in the Supplementary material.

### 2.5. Assessment of viral metagenomic content

Seawater was filtered through 0.2 μm polycarbonate membranes under low vacuum. The 0.2-μm filtrate was chemically treated with 1 mg L^−1^ FeCl_3_ to achieve viral particle flocculation within the following 6–12 h (John et al., 2011) and filtered again through 1 μm polycarbonate filters. The 1 μm filters were stored at 4°C, pending resuspension in ascorbic acid buffer. The solution of ascorbate-EDTA buffer was prepared daily (0.25 M ascorbic acid, 0.2 M Mg_2_EDTA, pH 6–6.5), kept in the dark and added in the viral flocculate, followed by hand shaking and overnight rotation at 4°C. After resuspension, viral particles in liquid were retained from the 1 μm filter by ultra-centrifugation at 141.000 g (SorvallTM WX100 ultracentrifuge, ThermoFisher Scientific, Sorvall TH 641 swing out rotor). Viral DNA was extracted following a CTAB protocol (Winnepenninckx et al., 1993) and purified as described in details elsewhere (Tsiola et al., 2020). The extracted DNA was dissolved in ultrapure water and stored at −20°C. Quantification of viral dsDNA was done with the Qubit high sensitivity assay kit in a 3.0 Qubit™ fluorometer (ThermoFisher Scientific).

Viral DNA shearing was done at 300 bp using the standard protocol for Covaris™ focused ultra-sonicator system. An indexed library for Illumina sequencing was prepared using the NEBNext Ultra DNA Library Prep Kit for Illumina (New England BioLabs) following the manual instructions. Size selection was done using AMPure XP beads (Beckman Coulter). PCR cycles were 6 (according to the manufacturer advice with regards to the amount of DNA input). Metagenomic libraries were sequenced in the Illumina Hiseq 4000 platform available at KAUST Bioscience Core Lab using paired-end sequencing.

Viral metagenome reads in FASTQ format were imported to CLC Genomics Workbench v.7 (CLC Bio) and trimmed using a minimum phred score of 20, a minimum length of 50 bp, allowing no ambiguous nucleotides and trimming off Illumina sequencing adaptors if found. The trimmed metagenome reads were assembled using CLC’s *de novo* assembly algorithm, using a k-mer of 63 and a minimum scaffold length of 500 bp. Raw reads were deposited on the NCBI with reference PRJNA996089.^1^ The assembled contigs were then analyzed using the iVirus pipeline (Bolduc et al., 2017) through the Cyverse platform (Goff et al., 2011) and VirFinder software (Ren et al., 2017) with criteria as described previously. Viral sequences with a VirFinder score ≥ 0.7 and *p* < 0.05 and VirSorter categories 1 and 2 were used for further analysis. The abundance and metabolic potential of auxiliary and metabolic potential of AMGs was determined through VIBRANT (v1.2.1; Kieft et al., 2019) using the virome option. The taxonomy of viral contigs was determined with the VPF-Class software which uses the IMG/VR v3 database for this purpose (Pons et al., 2021; Roux et al., 2021). Further, viral metagenomic sequence data were processed with the MetaPop multi-functional bioinformatic pipeline (Gregory et al., 2022) for macrodiversity analyses with the “vegan” R package. MetaPop was run with default parameters and cut-offs. Raw population abundances were normalized in order to mitigate sample-to-sample variation in the number of reads, via normalizing to the library with the highest number of reads. The abundance table of viral operational taxonomic units and then, it was used to generate a Bray-Curtis distance matrix. MetaPop macrodiversity outputs included population abundances, and alpha- (within community) and beta-diversity (between community) indices. Realizing the need to apply up-to-date pipelines for quality assessment of our viral metagenomes, we tested the clustering function of CheckV (Nayfach et al., 2021). However, no viruses were clustered indicating a lack of duplicates in our analyses. The raw number of viral metagenome reads, the quality of reads, the number of total contigs, the contig length (total, minimum and maximum) and the N50 contig length are summarized in Table 1.

**Table 1:** Raw numbers of viral metagenome reads, number of reads after quality control, number of total contigs, total contig length, the N50 contig length, and Shannon index of the viral metagenomes at the sampling stations.

Station	Depth (m)	Raw number of reads	Quality-controlled reads	Number of contigs	Total contig length (Mb)	N50 contig length (kb)	Shannon index
LV3	5	30,505,144	29,595,432	21,103	41,564	1,986	4,15
LV3	50	44,164,930	43,837,918	29,173	71,860	2,784	4,19
LV3	75	34,002,640	28,560,272	18,094	39,476	2,212	4,10
LV10	5	42,070,590	40,640,660	32,550	69,443	2,276	4,22
LV10	50	34,000,262	33,701,348	28,278	58,482	2,179	4,21
LV10	75	33,550,554	32,383,170	26,566	54,391	2,145	4,24
LV10	1,000	34,742,766	34,632,226	17,416	40,047	2,430	4,24
LV13	5	41,707,744	41,096,800	33,501	73,542	2,384	4,24
LV13	50	49,207,526	48,638,566	31,718	85,466	3,145	4,32
LV13	75	26,951,306	25,742,582	22,032	43,206	1,970	4,25
LV18	5	42,364,022	41,597,834	24,452	69,070	3,527	4,19
LV18	50	42,075,014	41,870,470	29,721	67,949	2,476	4,33
LV18	75	38,370,842	38,233,364	19,374	48,479	2,909	4,26
LV18	1,000	33,178,746	33,074,856	16,481	42,284	2,949	4,40

Samples from 1,000 m from stations LV3 and LV13 were not considered in this study since the raw number of reads was too low.

### 2.6. Statistics

Principal coordinates analysis was applied to coordinate the viral diversity and AMG data (Clarke and Ainsworth, 1993). Canonical analysis of principal coordinates (CAP) was applied to define clusters of samples based on “depth,” using the set of physical and chemical measurements (*S*, T, D, and the concentrations of PO_4_^3−^, NO_3_^−^, NO_2_^−^, NH_4_, SiO_4_, TN, TP, DO, DOC, DOP, DON). The clustering was tested by permutational multivariate analysis of variance (PERMANOVA). Then, patterns in viral diversity and AMG content between the stations were tested for significance by applying one-factor PERMANOVA using the factor “depth.” The null hypothesis was that there are no differences. When 5, 50, and 75 m were not different to each other, they were named as “surface/subsurface samples” for brevity. Bray–Curtis dissimilarity matrices on square-root transformed metagenomics data were constructed (Clarke and Warwick, 1994) to avoid misleading interpretations due to the sample-to-sample variation in read counts (Table 1). Hypothesis testing was performed using 999 permutations and pairwise tests using a significant level of 0.05. A principal coordinate analysis (PCoA) plot of all Bray-Curtis distances was created via the MetaPop pipeline and is presented as a means of macrodiversity visualization of the viral metagenomic data. The list of explanatory variables was normalized and the normalized matrix was used to create a resemblance matrix using Euclidean distances. Statistical analyses were done with the software package PRIMER v6 (PRIMER-E Ltd, Plymouth Marine Laboratory, Natural Environmental Research Council, United Kingdom) with PERMANOVA + add-on software (Anderson et al., 2008; Somerfield, 2008).

One-way analysis of variance (ANOVA) was applied to check for significant differences among the different depth layers in: the percentage contribution of individual genera and families over total viral contigs, the percentage contribution of viral AMGs over total number of AMGs and over their assigned KEGG category, the abundances of bacteria and virus-like particles, and finally in the physical, chemical and biological variables. The significance of the differences was assessed with *post hoc* Tukey test. Homogeneity of variance was checked using Levene’s test. ANOVAs were performed using IBM SPSS statistics software v23.

## 3. Results

### 3.1. Physical, chemical and biological features of the sampling area

**Figure 1:**
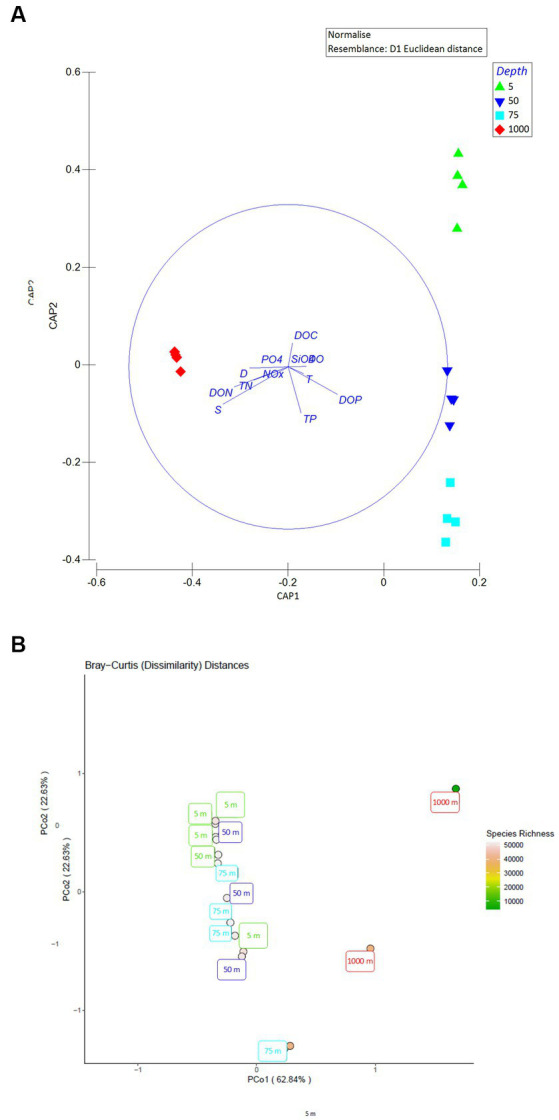
**(A)** Canonical analysis of principal coordinates (CAP) of the seawater physicochemical variables (blue solid-lined circle), depicting the sample grouping based on “depth” and Pearson correlations of the physicochemical variables with the canonical axes. Explanatory variables were normalized and the normalized matrix was used to create a resemblance matrix using Euclidean distances. S, salinity; T, temperature; D, density, concentrations of NOx, dissolved inorganic nitrogen; PO4, phosphate; DO, dissolved oxygen; SiO4, silicate; DON, dissolved organic nitrogen; DOC, dissolved organic carbon; TN, total nitrogen; and TP, total phosphorus. The length and direction of the vectors indicate their relative strength and direction of relationship, respectively. **(B)** Principal coordinates analysis (PCoA) ordination plot of all Bray-Curtis distances. An abundance table of viral operational taxonomic units was generated via MetaPop and then, the abundance table was used to generate a Bray-Curtis distance matrix to visualize viral community distributions using a PCoA ordination method. The color of each circle represents the species richness within each sample.

None of the measured variables differed between stations at the horizontal scale (PERMANOVA), thus, stations LV3, LV10, LV13, and LV18 are considered as replicates. Supplementary Table 1 summarizes output of all PERMANOVAs testing for differences between the sampling stations. Output of all PERMANOVAs testing variation among sampling depths are presented in Table 2, and not in the main body text (degrees of freedom, mean square, Pseudo-F ratio, *p* value). The Canonical Analysis of Principal coordinates (CAP) of physical and chemical variables (*S*, T, D, and the concentrations of NOx, PO_4_^3−^, DO, SiO_4_, DO, DOC, DOP, DON, TN and TP) revealed four significantly different groups, which were confirmed by PERMANOVA (*p* < 0.001). Samples from the same depth layers were grouped together and distantly from the others (Figure 1A).

**Table 2:** Output of permutational analysis of variance (PERMANOVA) testing variation among sampling depths.

Source of variation	df	MS	Pseudo-F	*p*
**Physicochemical variables: *S*, T, D, NOx, PO**_**4**_^**3−**^ **, SiO**_**4**_ **, DO, DOC, DOP, DON, TN, TP**				
Depth	3	51.27	23.48	0.001
Residuals	12	2.18		
**Abundances: virus-like particles, heterotrophic and autotrophic bacteria**				
Depth	3	313.94	15.45	0.001
Residuals	12	20.32		
**Viral community: family level**				
Depth	3	283.47	6.24	0.005
Residuals	10	45.44		
**Viral community: genus level**				
Depth	3	356.92	3.59	0.006
Residuals	10	99.48		
**Potential bacterial hosts of viruses**				
Depth	3	321.27	5.17	0.005
Residuals	10	62.16		
**AMG general metabolic pathways**				
Depth	3	906.46	4.32	0.009
Residuals	10	210.02		

Salinity (*S*), temperature (T), density (D), and the concentrations of phosphate (PO_4_^3−^), the sum of nitrate and nitrite (NOx), silicate (SiO_4_), total nitrogen (TN) and phosphorus (TP), dissolved oxygen (DO), dissolved organic carbon (DOC), phosphorus (DOP) and nitrogen (DON) and chlorophyll a (Chl) at the sampling stations.

*S* averaged 38.98 ± 0.13 in the sampling stations with equally high values at surface/subsurface depths (one-way ANOVA, *p* > 0.05). Approx. 0.21 lower *S* was measured at 1,000 m compared to averaged surface/subsurface values (Table 3) but the difference was significant only when 1,000 m were compared to 75 m (one-way ANOVA, *p* < 0.05). T ranged between 13.80°C and 18.30°C (Table 3). Small variation was seen between 50 and 75 m (16.81°C ± 0.46°C). All other depth layers exhibited significantly different T between them (one-way ANOVAs, *p* < 0.05), with the coldest waters seen at 1,000 m (*post hoc* Tukey test, p < 0.05).

**Table 3:** Salinity (*S*), temperature (T), density (D), and the concentrations of phosphate (PO_4_^3−^), the sum of nitrate and nitrite (NOx), silicate (SiO_4_), total nitrogen (TN) and phosphorus (TP), dissolved oxygen (DO), dissolved organic carbon (DOC), phosphorus (DOP) and nitrogen (DON) and chlorophyll a (Chl) at the sampling stations.

Station	Depth (m)	*S*	T	D	PO_4_^3−^	NOx	SiO_4_	TN	TP	DO	DOC	DOP	DON	Chl
		psu	^o^C	kg m^−3^	μM	μM	μM	μM	μM	mL L^−1^	μM	μM	μM	μg L^−1^
LV3	5	38.9	18.3	28.2	0.003	0.104	0.97	3.83	0.04	5.34	73	0.04	3.27	0.04
LV3	50	38.9	17.1	28.5	0.003	0.108	1.00	3.68	0.01	5.41	63	0.04	3.09	0.10
LV3	75	39.0	16.5	28.7	0.004	0.136	1.10	3.29	0.02	5.32	61	0.00	2.76	0.24
LV3	1,000	38.8	13.8	29.2	0.213	5.158	8.27	6.91	0.31	4.19	46	0.10	1.60	-
LV10	5	38.8	18.0	28.2	0.005	0.086	1.15	3.76	0.04	5.34	66	0.03	3.24	0.04
LV10	50	38.9	16.8	28.6	0.006	0.065	1.17	3.85	0.04	5.45	63	0.02	3.15	0.06
LV10	75	39.0	16.5	28.7	0.005	0.342	1.24	4.16	0.04	5.19	60	0.03	3.39	0.28
LV10	1,000	38.8	13.8	29.2	0.196	5.568	8.19	7.19	0.14	4.22	40	0.04	1.46	-
LV13	5	39.0	18.1	28.3	0.007	0.060	0.86	4.18	0.05	5.33	65	0.03	3.70	0.03
LV13	50	39.1	17.6	28.5	0.005	0.065	0.85	3.86	0.05	5.34	60	0.04	3.32	0.05
LV13	75	39.1	17.2	28.6	0.006	0.087	0.81	4.00	0.04	5.36	62	0.03	3.41	0.11
LV13	1,000	38.8	13.8	29.2	0.219	5.751	8.97	7.46	0.27	4.12	40	0.05	1.52	-
LV18	5	39.2	17.8	28.5	0.008	0.504	1.69	4.36	0.03	5.34	67	0.00	3.27	0.09
LV18	50	39.1	16.6	28.8	0.004	0.159	1.36	3.92	0.03	5.33	62	0.00	3.32	0.10
LV18	75	39.1	16.2	28.9	0.006	1.375	1.78	5.68	0.03	4.84	60	0.00	3.84	0.22
LV18	1,000	38.8	13.8	29.2	0.224	4.103	6.51	7.70	0.16	4.18	42	0.03	3.34	-

The concentrations of dissolved inorganic and organic nutrients significantly differed between surface/subsurface depths and 1,000 m (one-way ANOVAs, *p* < 0.05), being higher at 1,000 m (*post hoc* Tukey test, *p* < 0.05). DO concentration followed the opposite pattern (i.e., it was significantly lower at 1,000 m compared to surface/subsurface layers, *post hoc* Tukey test, *p* < 0.05). Measurements of these variables as well as of D, DOC, DON, DOP are presented in Table 3, and are discussed in the Supplementary material. Chl concentrations differed significantly between 5 and 75 m, and between 50 and 75 m (one-way ANOVAs, *p* < 0.05). Chl concentration was ~4x higher at 75 m than 5 m, and ~3x higher at 75 m than 50 m (*post hoc* Tukey tests, *p* < 0.05, Table 3).

### 3.2. Viral and bacterial abundances and production rates

**Figure 2:**
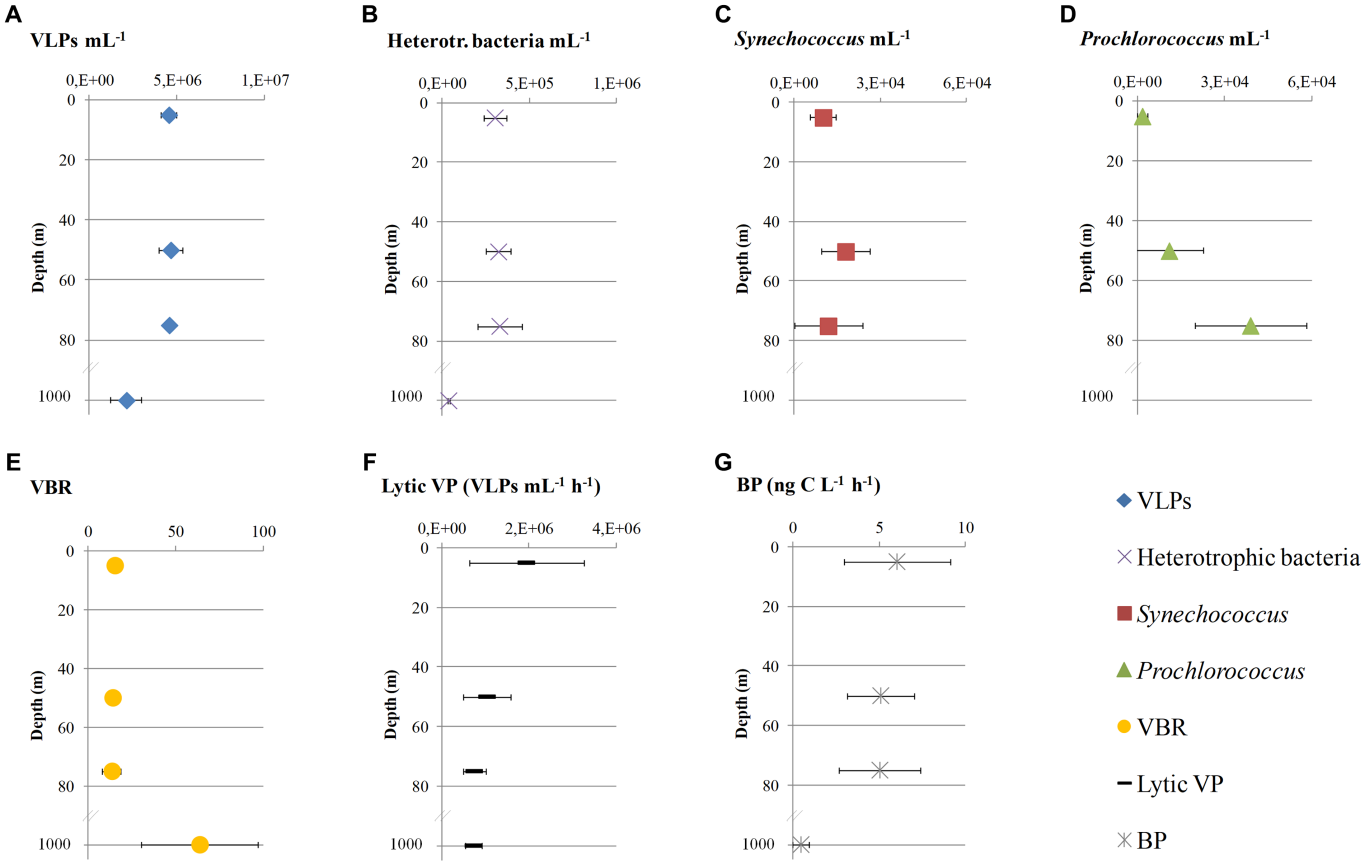
Abundances of virus-like particles (**A**: VLPs), heterotrophic bacteria **(B)**, *Synechococcus* **(C)**, *Prochlorococcus* **(D)**, the ratio between virus-like particles and total bacteria (**E**: VBR), lytic viral production rate (**F**: Lytic VP) and heterotrophic bacterial production (**G**: BP) at the sampling area represented as average values and standard deviations that derive from the four sampling stations (LV3, LV10, LV13, and LV18) at four sampling depths (5, 50, 75, and 1,000 m).

Neither viral (4.6 × 10^6^ ± 4.3 × 10^5^ mL^−1^) nor heterotrophic bacterial (3.2 × 10^5^ ± 8.4 × 10^4^ mL^−1^) abundances differed within the surface/subsurface layers (one-way ANOVAs, *p* > 0.05, Figures 2A,B). Significant differences were seen between surface/subsurface and 1,000 m samples at both cases (one-way ANOVA, *p* < 0.05); abundances were significantly lower at 1,000 m than at the other depths (*post hoc* Tukey test, *p* < 0.05). The abundance of *Synechococcus* did not differ between the sampling depths, opposite to *Prochlorococcus* (one-way ANOVA, *p* < 0.05), which was significantly lower at 5 than 75 m, as well as at 50 than 75 m (*post hoc* Tukey tests, *p* < 0.05). *Synechococcus* overcame in abundance *Prochlorococcus* by approx. one order of magnitude at 5 m, and the opposite was noticed at 75 m (Figures 2C,D). The resulting virus-to-bacteria ratio (VBR) varied greatly between surface/subsurface (9–21) and 1,000 m (56–107) considering in the calculation total bacterial abundances (Figure 2E). Significantly higher VBR was measured at 1,000 m than surface/subsurface samples (*post hoc* Tukey test, *p* < 0.05).

Lytic VP did not differ between the sampling depths (one-way ANOVA, *p* > 0.05), and it was slightly higher at 5 m (1.9 × 10^6^ ± 1.3 × 10^6^ cells mL^−1^ h^−1^) than the other depths (8.3 × 10^5^ ± 3.6 × 10^5^ cells mL^−1^ h^−1^; Figure 2F). BP did not differ within the surface/subsurface samples (one-way ANOVA, *p* > 0.05) and ranged between 2.35 and 9.28 ng C L^−1^ h^−1^ (Figure 2G). There was a significant difference between surface/subsurface and 1,000 m samples (one-way ANOVA, *p* < 0.05); significantly lower BP levels were seen at 1,000 m than surface/subsurface (*post hoc* Tukey test, *p* < 0.05).

### 3.3. Viral community composition

**Figure 3:**
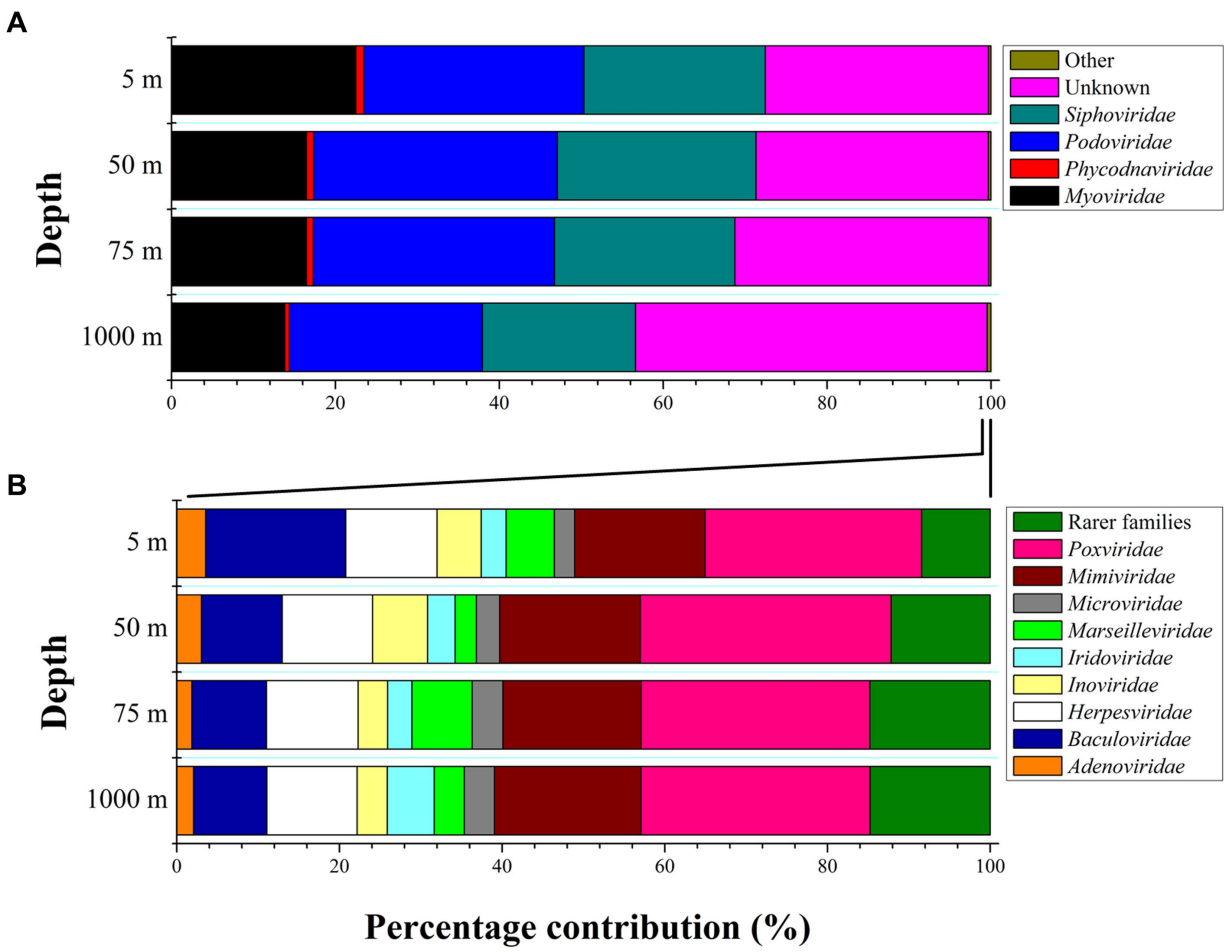
**(A,B)** Percentage contribution of the major viral families to total viral taxa at the sampling area represented as average values and standard deviations that derive from the four sampling stations (LV3, LV10, LV13, and LV18) at four sampling depths (5, 50, 75, and 1,000 m). “Other” families contribute <0.5% to the total reads and are expanded in the bottom left plot (at panel **B**: the 100% x axis refers to the “Other” fraction of panel **A**).

Viral community composition differed with the sampling depth at the family and genus level (PERMANOVAs, *p* < 0.01). Bray-Curtis distances were plotted using the principal coordinates analysis method (PCoA, Figure 1B). The two 1,000-m samples were more dissimilar to the rest and exhibited the highest species richness among all samples, while surface/subsurface samples were less dissimilar to each other and clustered together (Figure 1B). Alpha diversity indices (richness, Chao1, ACE, Shannon’s H, Simpsons, inverse Simpsons, Fisher, and Pielou’s J) for the samples are presented in Supplementary Table 1A. A percentage of 30 ± 7% of the contigs remained unassigned to the family level. Within the assigned contigs, the dominant families were *Podoviridae* (29% ± 3%) and *Siphoviridae* (23% ± 3%, Figure 3A). At 1,000 m, significantly lower contribution of *Podoviridae* (24% ± 2%) was seen compared to 5 m (27% ± 1%) and 50 m (30% ± 2%; one-way ANOVAs, *p* < 0.05). The same was noticed for *Siphoviridae* that contributed less at 1,000 m (19% ± 4%) compared to 5 m (22% ± 2%) and 50 m (24% ± 2%; one-way ANOVAs, *p* < 0.05). The third most abundant family was that of *Myoviridae*, exhibiting a decreasing trend (*post hoc* Tukey test, *p* < 0.05) from surface (23% ± 5% relative abundance) to deep waters (14% ± 0.3%, Figure 3A). *Phycodnaviridae* family was seen at all surface/subsurface stations with no significant differences in its contribution (0.7%–1.4%). The remaining taxa (<0.5%) were rare families (27 in total). Most of them were found in few stations each. The families *Adenoviridae*, *Baculoviridae*, *Herpesviridae*, *Inoviridae*, *Iridoviridae*, *Marseilleviridae*, *Microviridae*, *Mimiviridae*, *Poxviridae* were found in all stations (Figure 3B). The contribution of genera is presented in the Supplementary material (Supplementary text and Supplementary Table 2).

Information about the potential host assignment is presented in the Supplementary material.

### 3.4. Viral AMG content

**Figure 4:**
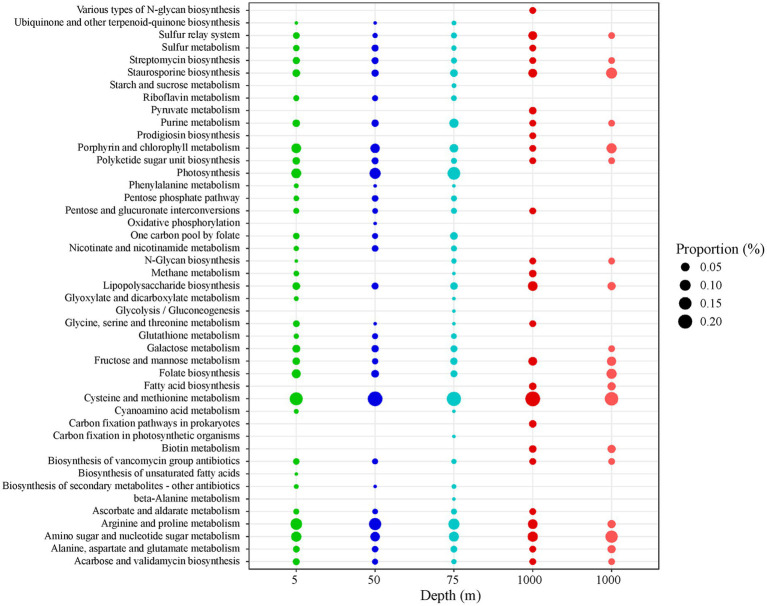
Bubble chart reporting the percentage contribution of the specific AMG metabolic pathways (as defined by KEGG using the VIBRANT method) over the total number of AMGs identified at the four sampling depths. For 5, 50, and 75 m: averaged values derive from the four sampling stations (LV3, LV10, LV13, and LV18). For 1,000 m, the values of the two deep stations are presented separately (dark red: LV10 and light red: LV18). The dimension of each bubble is proportional to the relative percentage contribution of the specific AMG categories.

A list of 143 AMGs was counted and associated with a metabolic pathway as defined by the Kyoto Encyclopedia of Gene (KEGG) using the VIBRANT method. The general pathways are shown in Table 4 and include: “amino acid metabolism,” “carbohydrate metabolism,” “metabolism of cofactors and vitamins,” “energy metabolism,” “lipid metabolism,” “glycan biosynthesis and metabolism,” “nucleotide metabolism,” “biosynthesis of secondary metabolites,” “folding, sorting and degradation,” “metabolism of other amino acids,” “metabolism of terpenoids and polyketides,” and “xenobiotics biodegradation and metabolism.” The specific metabolic pathways within the general ones are shown in Figure 4 and Supplementary Table 3. Statistical analysis for differences between the sampling depths was performed (1) in percentage contribution of specific AMG pathways within their general metabolic category, as well as (2) in percentage contribution of specific AMG pathways over total AMG reads.

**Table 4:** Percentage contribution of the general AMG metabolic pathways (as defined by KEGG using the VIBRANT method) identified at the sampling stations.

Station	Depth (m)	Amino acid	Carbohydrate	Cofactors and vitamins	Energy	Lipid	Glycan	Nucleotide	Secondary metabolites	Folding, sorting, degradation	Other amino acids	Terprenoids and polyketides	Xenobiotics
LV3	5	30.0	19.5	16.0	11.0	0.5	3.0	3.5	9.5	1.0	1.5	4.5	0
LV3	50	40.4	16.4	15.0	8.7	0	2.1	2.1	7.3	2.1	0.7	5.2	0
LV3	75	36.7	15.2	11.4	13.9	0	3.2	6.3	7.0	1.3	1.9	3.2	0
LV10	5	30.0	21.0	18.6	8.9	0.2	2.7	2.2	7.2	3.0	0.5	5.4	0.2
LV10	50	41.0	14.2	13.2	12.7	0.5	1.4	1.9	9.4	0.5	0.5	4.7	0
LV10	75	29.3	18.7	12.6	19.5	0.4	4.1	9.8	2.8	1.6	0.4	0.8	0
LV10	1,000	33.3	20.0	5.0	8.3	3.3	10	1.7	10.0	5.0	0	3.3	0
LV13	5	26.1	21.0	16.9	10.2	0.6	3.7	2.4	8.6	2.6	2.0	5.7	0.2
LV13	50	32.6	18.4	14.6	14.6	0.4	3.4	2.6	6.7	0.7	2.2	3.4	0.4
LV13	75	32.6	18.0	11.2	11.2	0	6.7	2.8	7.3	1.7	3.9	4.5	0
LV18	5	43.7	12.0	12.7	11.3	0	4.2	4.2	7.0	0.7	0.7	3.5	0
LV18	50	40.1	12.5	16.4	17.8	0	3.3	3.9	2.6	0	2.6	0.7	0
LV18	75	34.2	16.5	16.5	20.3	0	0	6.3	6.3	0	0	0	0
LV18	1,000	26.0	22.0	20.0	0	4.0	6.0	2.0	14.0	2.0	0	4.0	0

From left to right the full category description is: Amino acid metabolism, Carbohydrate metabolism, Metabolism of cofactors and vitamins, Energy metabolism, Lipid metabolism, Glycan biosynthesis and metabolism, Nucleotide metabolism, Biosynthesis of secondary metabolites, Folding, sorting and degradation, Metabolism of other amino acids, Metabolism of terpenoids and polyketides, Xenobiotics biodegradation and metabolism.

The content of AMGs differed with the sampling depth when considering both general and specific metabolic pathways (PERMANOVAs, *p* < 0.01). AMG content differed between 1,000 m and surface/subsurface samples. In terms of percentage contribution, the most frequently-detected general metabolic pathway was “amino acid metabolism” (34% ± 6%), followed by “carbohydrate metabolism” (18% ± 3%), “metabolism of cofactors and vitamins” (14% ± 4%), “energy metabolism” (12% ± 5%), and then “lipid metabolism” and “glycan biosynthesis and metabolism” (Table 4). The remaining categories are presented in the Supplementary material (Supplementary text and Supplementary Table 3).

#### 3.4.1. Amino acid metabolism AMGs

“Amino acid metabolism” AMGs were mostly related to “cysteine and methionine metabolism” and “arginine and proline metabolism” contributing 59% ± 8% and 32% ± 7% within the category, respectively (Supplementary Table 3). The “cysteine and methionine metabolism” AMGs contributed significantly less within the category at 5 m than the rest depths (one-way ANOVA, *p* < 0.05, *post hoc* Tukey test). The remaining percentage at 5 m was attributed to “glycine, serine and threonine metabolism” AMGs (Supplementary Table 3), involving *glyA*, *GATM* and *serA*. Within “cysteine and methionine metabolism,” the involved genes were *DNMT1*, *DNMT3A*, *mtnN*, *mtn* and *pfs* at all depths, while at 50 and 75 m the gene *yrrT* was also detected. The “arginine and proline metabolism” AMGs contributed significantly more within the category at 5, 50, and 75 m than 1,000 m (one-way ANOVA, *p* < 0.05, *post hoc* Tukey tests).

#### 3.4.2. Carbohydrate metabolism AMGs

Nearly half of the detected AMGs involved in “carbohydrate metabolism” were associated to “amino sugar and nucleotide sugar metabolism” (46% ± 8% within the category, Supplementary Table 3). “Amino sugar and nucleotide sugar metabolism” AMGs exhibited lower diversity at 1000 m (*ugd*, *gmd*, *ascC*, *ddhC*, *rfbH*, *algA*, *xanB*, *rfbA*, *wbpW*, *psIB*, *glmS*, *galE*) compared to surface/subsurface samples (all genes in the previous parenthesis and additionally: *TSTA3*, *fcl*, *per*, *rfbE*, *wecC*, *nagB*, *GNPDA*, *UDP-glucoronate 4-epimerase*, *GMPP*, *neuA*, *wbpP*, *glum*, *UDP-glucose 4,6-dehydratase*, *pgm* and *wbpA*). Other consistently-high “carbohydrate metabolism” AMGs were those involved in “fructose and mannose metabolism” having significantly higher percentage contribution over all AMGs at 1000 m than the rest depths (one-way ANOVA, *p* < 0.05, *post hoc* Tukey test, Figure 4). Higher diversity was also seen in genes involved at “fructose and mannose” in the surface/subsurface depths compared to 1,000 m. Opposite, “galactose metabolism” AMGs had significantly lower percentage contribution within the category at 1,000 m compared to the other depths (one-way ANOVA, *p* < 0.05, *post hoc* Tukey test, Supplementary Table 3). AMGs associated to “pentose phosphate pathway” (PPP) were not detected at 1,000 m. At surface/subsurface samples, PPP AMGs involved *rpiB*, *gnd*, *gntZ*, *PGD*, *zwf*, *pgm*, *talA* and *talB*. Only at 1,000 m, 2 reads assigned to “pyruvate metabolism” (*pps*, *ppsA*, Figure 4).

#### 3.4.3. Metabolism of cofactors and vitamins AMGs

Only at 1,000 m, AMGs related to “metabolism of cofactors and vitamins” included “biotin metabolism” (*fabG*, *fabF*, *fabZ*, Figure 4). At surface/subsurface depths, the detected AMGs were involved mainly in “porphyrin and chlorophyll metabolism” (*bchE*, *cobS*, *cobT,* and *ahbD*) and “riboflavin metabolism,” “folate biosynthesis” and “ubiquinone and other terpenoid-quinone biosynthesis” with highest contribution of “folate biosynthesis” (*folA*, *glyA*, *purT,* and *purN*) in all 5-m samples (Figure 4).

#### 3.4.4. Energy metabolism AMGs

Significantly higher contribution of “energy metabolism” AMGs was seen at 50 and 75 m compared to 1,000 m (one-way ANOVA, *p* < 0.05, *post hoc* Tukey tests, Table 4). More than 90% of “energy metabolism” AMGs were associated to “photosynthesis” at all 75 m-samples (Supplementary Table 3; Figure 4). The AMGs involved were *psbA* and *psbD*. At 5 and 50 m-samples, AMGs related to “photosynthesis” did not exceed 73% and 77%, respectively. At 5 and 50 m, AMGs were mostly associated to “sulfur metabolism” (20% ± 3%, *msmA*, *cysC*, *fccA,* and *dsrA*) and “oxidative phosphorylation” (*CYTB* and *petB*) and “methane metabolism” (*serA*, *PHGHD*, *glyA*, *SHMT*, *cofF*, *torD*, *fbaB*; Figure 4). At 1,000 m, “energy metabolism” AMGs were associated to “carbon fixation pathways in prokaryotes” (*pps* and *ppsA*, Figure 4) but also to “methane metabolism” and “sulfur metabolism” with different specific genes involved compared to surface/subsurface layers. At 1,000 m, *pps* and *ppsA* and *cysH* were found within “methane metabolism” and “sulfur metabolism,” respectively.

#### 3.4.5. Lipid metabolism AMGs

The percentage of “lipid metabolism” AMGs significantly differed between surface/subsurface depths and 1,000 m (one-way ANOVA, *p* < 0.05) being higher at 1,000 m (*post hoc* Tukey test; Table 4). All AMGs in the category were related to “fatty acid biosynthesis” at 1,000 m (Supplementary Table 3; Figure 4) involving *fabG*, *fabF,* and *fabZ*. In the surface/subsurface depths, no “fatty acid biosynthesis” AMGs were found and the great majority were related to “biosynthesis of unsaturated fatty acids” (*desC*; Figure 4).

#### 3.4.6. Glycan biosynthesis and metabolism AMGs

The percentage of “glycan biosynthesis and metabolism” AMGs significantly differed between surface/subsurface depths and 1,000 m (one-way ANOVA, *p* < 0.05) being higher at 1,000 m (*post hoc* Tukey test; Table 4). Most AMGs were related to “lipopolysaccharide biosynthesis” (Figure 4), with the most common AMGs at 1,000 m being *waaF*, *rfaF* and *rfaE1* and *lpxA*, and at surface/subsurface depths being *kdsB*, *kdsA*, *lpxH*, *gmhC*, and *gmhD*.

## 4. Discussion

### 4.1. Same lysis rate at all depths—different taxa and AMGs involved

We studied oceanic viral populations taking into consideration the lytic efficiency and the possible mechanisms that viruses employ to alter their hosts’ metabolism using their own metagenome footprint. Our results demonstrate clear depth-dependent patterns (surface/subsurface shallow vs. deep waters) in viral community composition and AMG content during the early stratification season in the Eastern Mediterranean Sea. The vertical environmental variability of this oligotrophic basin was considered in order to uncover, and propose viral actions that lead to energy acquisition and lysis sustenance. The depth-dependent patterns were linked to differences in temperature, nutrient availability (total and dissolved nitrogen and phosphorus), host productivity and host population density. While viral productivity levels did not change significantly along the water column, the numbers of free viruses per bacteria (VBR) were significantly higher at 1,000 m, resembling a similar peak in VBR in shallower mesopelagic depths in the same study area (Magiopoulos and Pitta, 2012). Interestingly, the diversity of viral taxa and AMGs was also substantially different at this depth. The presented findings suggest that a similar quantitative effect of viruses on their hosts (lytic activity) is achieved by different viral populations carrying different auxiliary metabolic genes.

Differences in viral communities with temperature were highlighted in the Mediterranean and globally (Coutinho et al., 2019; Gregory et al., 2019) and differences with depth were also reported for the hadal zone (Jian et al., 2021) and the Pacific Ocean using either high-throughput sequencing technology (Hurwitz et al., 2015) or genome fingerprinting approaches (Steward et al., 2000; Brum, 2005). Coutinho et al. (2019) have reported a peak in the Shannon diversity index in deep water of the western-Mediterranean. Lower contribution of the dominant families in deep rather than surface waters was seen in the hadal zone of Challenger Deep (Gao et al., 2022). Similarly, the highest species richness was recorded at 1,000 m, with the community encompassing less *Podoviridae* and *Siphoviridae* members but more unassigned reads, which is reasonable as a more complex community may sustain and optimize ecosystem functioning under low-oxygen and low-nutrients conditions.

Additionally, at 1,000 m we found that the AMG content was different than at the other depths. The main distinctive features of the deep layers in comparison to the shallower ones were (a) a higher contribution of genes involved in the sulfur relay system, the synthesis and metabolism of glycan and the metabolism of fructose, mannose, fatty acids, cofactors and vitamins and secondary metabolites, (b) a lower contribution of genes involved in energy metabolic pathways, and the metabolism of arginine, proline and galactose, (c) different AMG content within the categories of metabolism of amino sugar and sugar nucleotide, fructose and mannose, lipopolysaccharide, sulfur and methane, lipids and purine, and finally (d) the absence of genes related to pentose phosphate pathway, and the unique finding of genes related to pyruvate and biotin metabolism, and prodigiosin biosynthesis. At this point, we urge the readers to take into consideration the low number of replicated samples when deepening to our findings. Consideration of seasonal and inter-annual changes in irradiance, temperature and nutrient levels with depth would have also improved our exploration, since it is widely known that these changes largely impact viral activity (Brum et al., 2016; Coutinho et al., 2017; Gainer et al., 2017; Puxty et al., 2018).

In the energy-limited bathypelagic zone, it is expected that viruses, as their hosts (Konstantinidis et al., 2009), need to have higher metabolic flexibility and thus, exploit a variety of AMGs to support their reproduction in comparison to the more limited resources. Recent reports consider that viral genomes are under the selective pressure of the environment (Heyerhoff et al., 2022); in natural populations, the availability of nitrogen (Jian et al., 2021) and phosphorus (Kelly et al., 2013) and the variable temperature conditions (Coutinho et al., 2019) seem to drive differences in viral genomes from the community to the protein level. While the total amount of AMGs entailed in energy acquisition was lower at 1,000 m compared to surface/subsurface waters, the particular genes involved were rather more variable and seemed to ensure that diverse substrates and energy sources can be used in the EMS deep environment where light, organic matter and nutrients are limited and the growth of bacteria is carbon-limited (Rahav et al., 2019). In the bathypelagic realm of the oligotrophic EMS, nutrients are scattered in time and space, thus their high demand may have caused relevant viral genome adaptions that are discussed in chapters 4.2. and 4.3.

### 4.2. Diversion of host metabolism toward gluconeogenesis, fatty-acid and glycan biosynthesis and metabolism, and sulfur relay may support lytic activity in the deep EMS

A higher contribution of AMGs related to energy production via gluconeogesis (*pps*, *ppsA*) was seen in the deepest samples, suggesting that the use of alternative carbon sources for the synthesis of glucose and polysaccharides is positively selected at 1,000 m. In deep waters, hexoses are preferentially removed from high-molecular weight DOC by bacteria (Garel et al., 2021). The number of hexose metabolism genes was higher in the deep, and this fact led us to think that viruses may provide a vital mechanism to build the precursors for ATP synthesis and aid viral proliferation, thus supporting the high microbial respiration needs in the bathypelagic zone (Acinas et al., 2021). AMGs involved in the pentose phosphate pathway (PPP) was proposed to divert host metabolism to nucleotide synthesis and energy production (Thompson et al., 2011). PPP is probably not boosted in the bathypelagic zone in our study area but this hypothesis needs confirmation using a larger set of samples and replicates. A range of PPP genes (*rpi*, *gnd*, *zwf*) was seen in the surface/subsurface samples (but not in the deep) where light and energy availability is not a limiting step for host and viral productivity, similar to recent observations for surface coastal waters of the Mediterranean (Tsiola et al., 2020).

A higher contribution of AMGs related to fatty-acid biosynthesis was seen at 1,000 m, suggesting that the elongation of fatty acids is promoted through viral AMGs. *fabG* encodes for the NADPH-dependent reduction of beta-ketoacyl-ACP substrates to beta-hydroxyacyl-ACP products, and has been found in viromes in the hadal zone (Jian et al., 2021). Alongside, the reaction of the enzyme of *fabZ* was a bottleneck in the fatty-acid production in numerous microbial hosts (Jeon et al., 2012). The presence of *fabG* and *fabZ* in the deep may be related to the need of fatty acids as electron donors in several bacteria. Sulfate-reducing bacteria for example are optimized to fully oxidize fatty acids; interestingly, their presence was indicated by the concomitant detection of *CysH* gene at 1,000 m.

The higher contribution of AMGs involved in the glycan biosynthesis in the deep supports the scenario of accelerated viral folding and structuring in these systems, something that totally agrees with the high VBR and VP lytic measurements in our study. The identification of genes involved in the amino sugar and nucleotide sugar metabolism along with genes involved in glycoprotein synthesis and metabolism (*waaF*, *rfaE*; now known as *hldE*, having key roles in the biosynthetic process of lipopolysaccharides, Graves et al., 2001; Weynberg et al., 2017) is a unique coupled finding for deep waters, that confirms the magnitude of hosts-gene hijacking in natural populations. In addition, the different repertoire of AMGs at the four depths involved in glycan metabolism, thus in correct host recognition, attachment and entry to the bacterial host cell (Kortright et al., 2020). indicates that attachment and penetration to hosts may happen by exploiting different strategies in deep vs. shallow waters, ultimately leading to equally high lytic activity at all depth layers.

The higher contribution of the *mec* gene at 1,000 m seems to assist nutrient acquisition in this deep ecosystem, since the gene encodes for a sulfur carrier protein that aids the biosynthesis of cysteine (Hesketh-Best et al., 2022) and possibly the degradation of sulfated organic matter (Han et al., 2023), thus viral particle formation too. Despite the low understanding of the role of *mec* in the viral genomes, it seems that recent investigations on viral implications on the oceanic sulfur cycling are reinforced and extended by our findings (Kieft et al., 2021); viral genes related to sulfur relay were found at 1,000 m, thus possibly contributing to sulfur’s budget manipulation.

We propose that the various above-mentioned mechanisms may have been adapted by the viral populations in order to more efficiently use the scattered energy sources in the oligotrophic deep part of the EMS water column. In such case, the selective advantage of viruses can reasonably justify the high VBR and lytic production in all depth layers, despite the low nutritional status of the bathypelagic zone.

### 4.3. Change of amino-acid frequency aids adaptation to the cold in the deep

Arginine and proline metabolism AMGs contributed less in the deepest samples, confirming that viruses “select” for certain amino acids for tolerance to cold temperatures (Alarcón-Schumacher et al., 2021) and optimal capsid formation (Weynberg et al., 2017).

### 4.4. Surface (5 m) vs. subsurface (50 and 75 m) patterns in AMG content

The most noticeable difference in the AMG content between between 5, 50, and 75 m was that of the DCM; at 75 m, photosynthesis-related *psbA* and *psbD* outnumbered the energy-metabolism AMGs (>90%), while at 5 and 50 m these genes were significantly less. Indeed, comparing with *in situ* primary production measurements (Livanou et al., 2019; Psarra unpubl. data), primary production maxima at subsurface layers were consistently recorded just above the DCM layer. It was found that the *psb* genes are expressed during the viral latent period, thus their overrepresentation in the DCM seems supportive of extensive photosynthesis at this layer (Sharon et al., 2007; Sieradzki et al., 2019). At 75 m, no energy-production related to sulfur seemed to occur through viral AMGs, and photosynthesis governed. On the contrary, at 5 and 50 m, a repertoire of viral AMGs related to sulfur exploitation either for ATP production or for sulfur assimilation and biosynthetic processes was found; dissimilatory (e.g., *dsrA*) and assimilatory (e.g., *cysC*) sulfur metabolism genes support the auxiliary metabolism of sulfur in the host cells (Kieft et al., 2021). The *dsrA*, encoding for dissimilatory sulfur reductase within the pathway of sulfur oxidation was noted in the latter study, while Roux et al. (2016) and references therein have mentioned only the dissimilatory sulfur reductase subunit C (*dsrC*) in various pelagic and benthic samples. The authors proposed that viruses assist their sulfur oxidizer hosts through the *dsrC*-like genes under nutrient limiting conditions (Roux et al., 2016) that could be valid in the oligotrophic conditions of the studied area. Our findings are in agreement with previous observations that viral diversity does not differ substantially between surface and deep-chlorophyll maximum (DCM) layers except when considering protein clusters (Brum et al., 2015). An additional indication that excessive energy at DCM was diverted toward viral replication is the peak of purine metabolism genes (Puxty et al., 2015) at 75 m.

### 4.5. Conclusion

A comprehensive description of viral dynamics in surface (5 m), subsurface (50 and 75 m) and deep (1,000 m) off-shore waters of the EMS was achieved by combining whole viral metagenome sequencing, flow cytometric and productivity analyses, assuming that such a combination is the foundation of meaningful viral ecology discussions. The findings confirm our hypothesis that a more diverse repertoire of AMGs is present in bathypelagic than shallow EMS waters, diverting hosts’ metabolism toward energy production and thus, viral replication too. Further work is needed to generalize our implications to other ecosystems and seasons with regards to the diel, seasonal and annual variations in irradiance, temperature and other physicochemical characteristics of the water masses, since the sampling power in this study was limited by field-work constraints and analytical costs. AMG content was substantially different between 1,000 m and the shallower layers, revealing novel implications for the energy-production mechanisms in the resource-depleted deep waters of the EMS. The lysis rate was, however, similar along the vertical profile. Altogether, our findings advocate that viral interference with hosts’ metabolism is affected by the particular environmental setting, and must be carefully considered for deciphering the biogeochemical turnover in the oceans.

## Data availability statement

The metagenomic datasets presented in this study can be found in online repositories. The names of the repository/repositories and accession number(s) can be found in the article Section 2.5.

## Author contributions

AT: Conceptualization, Data curation, Formal analysis, Writing – original draft, Writing – review & editing. GM: Data curation, Software, Writing – review & editing, Methodology. DD: Funding acquisition, Supervision, Writing – review & editing. SF: Methodology, Writing – review & editing. AG: Methodology, Writing – review & editing. DM: Methodology, Writing – review & editing. AP: Methodology, Writing – review & editing. EP: Methodology, Writing – review & editing. SP: Conceptualization, Data curation, Methodology, Project administration, Writing – review & editing. IS: Data curation, Methodology, Writing – review & editing. CZ: Methodology, Writing – review & editing. PP: Funding acquisition, Supervision, Writing – review & editing.

## Footnotes

^1^ https://www.ncbi.nlm.nih.gov/sra/PRJNA996089
