# Supplementary material for: Depth-driven patterns in lytic viral diversity, auxiliary metabolic gene content, and productivity in offshore oligotrophic waters
Source: Front Microbiol. 2023 Nov 2;14:1271535. doi: 10.3389/fmicb.2023.1271535 (PMC10653327; doi:10.3389/fmicb.2023.1271535)
Supplement: Supplementary file 1 [file Data_Sheet_1.pdf]

Supplementary Material

Depth-driven patterns in lytic viral diversity, auxiliary metabolic gene content, and productivity in offshore oligotrophic waters

Anastasia Tsiola*, Grégoire Michoud, Daniele Daffonchio, Stilianos Fodelianakis, Antonia Giannakourou, Dimitris Malliarakis, Alexandra Pavlidou, Elli Pitta, Stella Psarra, Ioulia Santi, Christina Zeri, Paraskevi Pitta

*** Correspondence:** Tsiola Anastasia: atsiola@hcmr.gr

1 Supplementary Data

Materials and Methods

Assessment of physical and chemical characteristics

The determination of the dissolved oxygen (DO) concentration was performed on board immediately after seawater sampling. Seawater samples were collected from the Rosette-Niskin sampling system with the typical precaution to prevent any biological activity and gas exchange with the atmosphere (Strickland and Parsons, 1972). Special Winkler glass bottles with beveled glass stoppers and measured capacity specified to ±0.01 mL were used. Chemical reagents were added immediately after sampling. The determination of DO was conducted by titration using the Winkler method according to Carpenter (Carpenter, 1965). The titration was carried out with a standardized thiosulphate solution using a Metrohm 876 Dosimat (Switzerland). Quality control/assurance was accomplished through the standardization of the thiosulphate solution with a reference standard solution of potassium iodide on a daily basis. The precision of the method outlined above is estimated to be 2.2 μmol/L.

Samples for the determination of nitrate, nitrite, silicate were kept frozen until analysis in the certified according to ISO 17025 biogeochemical laboratories of HCMR. Samples were not filtrated to avoid contamination. Nitrate, nitrite (Strickland and Parsons, 1972), phosphate (Murphy and Riley, 1962) and silicate (Mullin and Riley, 1955) concentrations were measured on a Seal III autoanalyzer according to standard methods. Total nitrogen (TN) and total phosphorus (TP) concentrations were measured following a wet-oxidation with persulfate in low alkaline conditions. After oxidation, the concentration of the inorganic products dissolved in the sample is measured automatically by colorimetry using a SEAL III autoanalyser (Pujo-Pay and Raimbault, 1994; Raimbault et al., 1999). The concentration of dissolved organic nitrogen [DON] was calculated by subtracting the sum of dissolved inorganic nitrogen ([DIN] = [NO3-+NO2-] + [NH4+]) and the particulate nitrogen [PN] from the measured [TN]; [DON] = [TN]–[DIN]-[PN]. The concentration of dissolved organic phosphorus (DOP) was calculated by subtracting phosphates and particulate phosphorus (PP) from the measured [TDP]; [DOP] = [TP]–[PO43-]-[PP]. The values obtained were corrected for the reagent blank.

The determination of ammonium and phosphate was performed on board using a 25Lambda Perkin Elmer spectrophotometer (Koroleff, 1970). Sensitive (nanomolar) technology was used for phosphate determination. Phosphate concentration in the upper 300 m of the water column was measured using the "magic" method (Rimmelin and Moutin, 2005). Phosphate concentration in the deeper layer 300-bottom was measured using the "classic" method for phosphate analysis based on Murphy and Riley (Murphy and Riley, 1962) standard method. The limits of quantification for the methods used are: 0.152 μmol/L for nitrate+nitrite; 0.025 μmol/L for nitrite; 0.274 μmol/L for silicate; 0.102 μmol/L for ammonium and 0.010 μmol/L for phosphate analysis.

Dissolved organic carbon (DOC) concentrations were determined using a Shimadzu TOC 5000A organic carbon analyzer and following the high temperature catalytic oxidation method (HTCO) as described by Sugimura and Suzuki (Sugimura and Suzuki, 1988) and Cauwet (Cauwet, 1994). The system was standardized prior to analysis using a potassium hydrogen phthalate standard solution series. Analytical precision and accuracy were tested against Deep Atlantic Seawater Reference Material provided by the DOC-CRM program (University of Miami – D.A. Hansell, batch 13 - reference value 41-43 μmol L^{-1}, measured values 42-43 μmol L^{-1}).

Chlorophyll a (Chl) concentration was determined by High Performance Liquid Chromatography (HPLC) analysis in the euphotic layer down to 120 m. Two liters of seawater were filtered through GF/F filters (25 mm) under low vacuum pressure (<150 mmHg). The complete analysis description and the applied chromatographic conditions are described in Lagaria et al. (Lagaria et al., 2017).

Assessment of heterotrophic bacterial production rates

Heterotrophic Bacterial Production (BP) was measured with the [^{3}H] leucine incorporation method (Kirchman et al., 1986) as modified by Smith and Azam (1992). At each depth 1,5 mL duplicate samples and one trichloracetic acid (TCA) killed control were spiked with L-[4.5-3H] leucine (Perkin Elmer, 115 Ci/mmol) at final concentration of 20 nM. All samples, including controls, were incubated for 2 h in the dark and at *in situ* temperature. Incubations were stopped with 90 μl of 100% TCA and the samples were stored at 4°C in the dark until further processing. Centrifugation was carried out at 16000 g for 10 min. After discarding the supernatant, 1.5 ml of 5% TCA was added, the samples were vigorously shaken using a vortex and then centrifuged again. After discarding the supernatant, 1.5 ml of 80% ethanol was added, the samples were shaken again and centrifuged. The supernatant was then discarded, and 1.5 ml scintillation liquid was added (Ultima Gold-Packard). The radioactivity incorporated into the pellet was counted using a Packard LS 1600 Liquid Scintillation Counter. A time series experiment was carried out in order to show that the incorporation of leucine was linear with time. A concentration kinetic experiment was also performed to verify that the concentration of leucine added (20 nM) was sufficient to saturation. Incorporation rates of leucine into carbon were calculated using a theoretic conversion factor of 1.55 kg of C mol^{-1} (Kirchman 1993).

References

Carpenter, J. H. (1965). The accuracy of the Winkler method for dissolved oxygen analysis. *Limnol. Oceanogr.* 10, 135–140.

Cauwet, G. (1994). HTCO method for dissolved organic carbon analysis in seawater: influence of catalyst on blank estimation. *Mar. Chem.* 47, 55–64. doi: 10.1016/0304-4203(94)90013-2.

Kirchmann D.L. (1993). Leucine incorporation as a measure of biomass production by heterotrophic bacteria. *In:* PF Kemp, BF Sherr, EB Sherr, JJ Cole (eds), Handbook of Methods in Aquatic Microbial Ecology. Lewis, Boca Raton, pp 509-512.

Kirchman, D., Newell, S., and Hodson, R. (1986). Incorporation versus biosynthesis of leucine: implications for measuring rates of protein synthesis and biomass production by bacteria in marine systems. *Mar. Ecol. Prog. Ser.* 32, 47–59. doi: 10.3354/meps032047.

Koroleff, F. (1970). Revised version of "Determination of ammonia in natural waters as indophenol blue." *Interlab Rep* ICES infor, 19–22.

Lagaria, A., Mandalakis, M., Mara, P., Frangoulis, C., Karatsolis, B.-T. T., Pitta, P., et al. (2017). Phytoplankton variability and community structure in relation to hydrographic features in the NE Aegean frontal area (NE Mediterranean Sea). *Cont. Shelf Res.* 149, 124–137. doi: 10.1016/j.csr.2016.07.014.

Mullin, J. B., and Riley, J. P. (1955). The colorimetric determination of silicate with special references to sea and natural water. *Anal. chim. Acta* 12, 162–176.

Murphy, J., and Riley, J. P. (1962). A modified single solution method for the determination of phosphate in natural waters. *Anal. Chim. Acta 31* 27, 31–36. doi: 10.1016/S0003-2670(00)88444-5.

Pujo-Pay, M., and Raimbault, P. (1994). Improvement of the wet-oxidation procedure for simultaneous determination of particulate organic nitrogen and phosphorus collected on filters. *Mar. Ecol. Prog. Ser.* 105, 203. doi: 10.3354/meps105203.

Raimbault, P., Diaz, F., Pouvesle, W., and Boudjellal, B. (1999). Simultaneous determination of particulate organic carbon, nitrogen and phosphorus collected on filters, using a semi-automatic wet-oxidation method. *Mar. Ecol. Prog. Ser.* 180, 289–295.

Rimmelin, P., and Moutin, T. (2005). Re-examination of the MAGIC method to determine low orthophosphate concentration in seawater. *Anal. Chim. Acta* 548, 174–182. doi: 10.1016/j.aca.2005.05.071.

Smith D.C., Azam F. (1992). A simple, economical method for measuring bacterial protein synthesis rates in sea water using 3H-leucine. Mar Microb Food Webs 6: 107-114.

Strickland, J., and Parsons, T. (1972). "Determination of phosphorus," in *A practical handbook of sewater analysis*, 49.

Sugimura, Y., and Suzuki, Y. (1988). A high-temperature catalytic oxidation method for the determination of non-volatile dissolved organic carbon in seawater by direct injection of a liquid sample. *Mar. Chem.* 24, 105–131. doi: 10.1016/0304-4203(88)90043-6.

Results

Physical, chemical and biological features of the sampling area

The concentrations of phosphate (PO_4^{3-}), the sum of nitrate and nitrite (NOx) and silicate (SiO_4) significantly differed between surface/subsurface (5, 50 and 75 m) and 1000 m samples (one-way ANOVA, $p<0.05$). In specific, PO_4^{3-} concentrations were lower at the surface/subsurface than at 1000 m (post hoc Tukey test, $p<0.05$). Nearly undetected levels (<0.01 μM) were measured at the former stations and 0.21±0.01 μM at the latter (Table 1). Similarly, NOx concentrations were ~20x higher at 1000 m compared to the surface/subsurface layers (post hoc Tukey test, $p<0.05$), reaching maximum values, >5 μM (Table 1). In addition, SiO_4 concentrations were significantly higher (~7x) at 1000 m compared to all other samples (post hoc Tukey test, $p<0.05$).

Total nitrogen (TN) and total phosphorus (TP) concentrations followed the same pattern as the dissolved inorganic nutrient forms; both concentrations were significantly different between surface/subsurface (5, 50 and 75 m) and 1000 m samples (one-way ANOVA, $p<0.05$). In specific, the concentrations at 1000 m were ~2x higher for TN (maximum 7.70 μM at LV18) and ~5x higher for TP (maximum 0.27 μM at LV13, Table 1) compared to the other waters masses (post hoc Tukey test, $p<0.05$). The concentrations of dissolved organic carbon (DOC) differed between all sampling depths (one-way ANOVA, $p<0.05$) except for 50 and 75 m where it ranged minimally between 0.060 and 0.063 μmol L^{-1} (Table 1). At 5 m, DOC was significantly higher compared to the other depths (post hoc Tukey test, $p<0.05$) exhibiting maximum value at LV3 (0.073 μmol L^{-1}). On the other hand, DOC was significantly lower at 1000 m compared to the other depths (post hoc Tukey test, $p<0.05$). Opposite to DOC, dissolved organic nitrogen (DON) concentrations differed only between the surface/subsurface (5, 50 and 75 m) and 1000 m samples (one-way ANOVA, $p<0.05$). Significantly lower values (~2x lower) were measured at 1000 m (post hoc Tukey test, $p<0.05$) compared to the other depths (Table 1). DOP values were very low at all stations (~ 10^{-5} μmol L^{-1}).

The concentration of dissolved oxygen (DO) varied little at surface/subsurface (5.30±0.16 mL L^{-1}, Table 1) and was significantly different from the concentration measured at 1000 m (one-way ANOVA, $p<0.05$). DO levels at 1000 m were ~4x lower (between 4.12 and 4.22 mL L^{-1}, Table 1) than at the surface/subsurface (post hoc Tukey test, $p<0.05$).

Density (D) averaged 28.64±0.32 kg m^{-3} in the sampling stations (Table 1). Similarly to T, density was not different between 50 and 75 m but it was significantly different between all other depth layers (one-way ANOVAs, $p<0.05$). In specific, D was higher at 1000 m compared to surface/subsurface depths (post hoc Tukey test, $p<0.05$) by approx. 0.66 kg m^{-3}.

Viral community composition

The most dominant genera were *Lambdavirus* and *T4virus*. The contribution of *Lambdavirus* at 5, 50 and 75 m ranged between 22 and 29% (Supplementary Table 2). At 1000 m, *Lambdavirus* contribution was significantly different than the other depths (one-way ANOVA, $p<0.05$). Specifically, it was lower (post hoc Tukey test, $p<0.05$; 23 at LV13 and 15% at LV18, respectively). Similarly, *T4virus* contributed significantly more at 5 m than 1000 m (one-way ANOVA, post hoc Tukey test, $p<0.05$). In contrast, *T4virus* contribution did not exceed 13% at 1000 m. *T4virus* contribution between 50 m and 75 m was indifferent. Similarly to *T4virus*, the genus *M12virus* reached significantly higher contribution at 5 m compared to 1000 m only (one-way ANOVA, post

hoc Tukey test, $p<0.05$), i.e. up to 9% of the total contigs. *Septima3virus* contributed equally at all stations (2±0.3%), similarly to *Vp5virus* (3±1%) and *Bcep22virus* (2±0.4%). *Bpp1virus* contributed up to 2% at all surface/subsurface stations but it was not found at 1000 m (one-way ANOVA, post hoc Tukey test, $p<0.05$). Among the rest of the detected genera, *Chlorovirus*, *Cp220virus*, *Kp36virus*, *Muvirus*, *P70virus*, *Prtbvirus*, *Prymnesiovirus*, *Slashvirus*, *Vegasvirus* were found in at least one station having >2% contribution, while the genera *Cba41virus*, *Luz24virus*, *Nit1virus*, *P12002virus*, *P12024virus* were found at all stations having >2% contribution (Supplementary Table 2).

Based on the SIMPER test, samples from 50 m were dissimilar from 5, 75 and 1000 m by 9, 10 and 23%, respectively. For the first case, the most responsible taxon for the 9% dissimilarity was *Myoviridae* (27% contribution), while the dissimilarity between 50 m and either 75 or 1000 m was mostly attributed to *Podoviridae* (18 and 27% contribution, respectively). Also based on the SIMPER test, samples from 1000 m differed from 5, 50 and 75 m samples by 22, 23 and 18%, respectively. For the first case, the most responsible taxon for the 22% dissimilarity was *Myoviridae* (28% contribution), while at the other two cases, the most responsible taxa for the 23 and 18% dissimilarities was *Podoviridae* (27 and 30% contribution, respectively).

Viral potential host assignment

A total number of 72 bacterial families were assigned as potential hosts for the lytic viral contigs. PERMANOVA showed that potential host community composition differed with the sampling depth (PERMANOVA, $p<0.01$). Additional information is presented in the Supplementary Material. All depths were clustered separately from each other, with the surface/subsurface and 1000 m samples showing >20% dissimilarity (SIMPROF test). The most dominant potential hosts were *Synechococcaceae* (19-25% at surface/subsurface) and *Enterobacteriacea* (19-25%) as well as *Flavobacteriacea* (5-8% at surface/subsurface, 3% at 1000 m and *Vibrionaceae* (3-5%) (Supplementary Figure 1). The most dominant potential hosts were *Synechococcaceae* (19-25% at surface/subsurface) and *Enterobacteriacea* (19-25%) as well as *Flavobacteriacea* (5-8% at surface/subsurface, 3% at 1000 m and *Vibrionaceae* (3-5%) (Supplementary Figure 1). The contribution of these families was significantly between surface/subsurface and 1000 m (one-way ANOVA, $p<0.05$). Hosts that could not be affiliated to the family level (unknown) constituted 30, 31, 33 and 45% of the reads at 5, 50, 75 and 1000 m, respectively (Supplementary Figure 1). Additional information is presented in the Supplementary Material.

A mild (not significant) depth-driven pattern was seen for *Bacillaceae* that contributed between 2 and 3% at all stations except 1000 m where this range was 4 to 6% (LV10 and LV18, respectively). *Pseudomonadaceae* contribution also increased mildly with depth (1±0.1% at 5 m, 2±0.2% at 50, 2±0.1% at 75 m, 3±0.4% at 1000 m). In contrast, the contribution of *Rhodothermaceae* decreased with depth (3±1 at 5 m, 2±0.1 at 50 m, 2±0.4 at 75 m, 1±0.2 at 1000 m) with the difference between 5 and 1000 m being significant (one-way ANOVA, post hoc Tukey test, $p<0.05$). *Prochloraceae* was not found at 75 m and 1000 m and it contributed 1±0.2% at 5 m and 50 m. Additionally, *Mycobacteriaceae* (1±0.1%), *Rhizobiacea* (1±0.3%), *Burkholderiacea* (2±0.2%) and *Haloarculaceae* (0.4-1% that showed highest percentages at LV13 and LV18) were present at all stations (Supplementary Figure 1).

Viral AMG content

Nucleotide metabolism

"Nucleotide metabolism" AMGs were at all cases associated to "purine metabolism". Significantly higher percentage contribution of this category was seen at 75 m compared to 1000 m and 50 m (one-way ANOVA, $p<0.05$, post hoc Tukey tests). Within surface/subsurface layers, a wide variety of "nucleotide metabolism" AMGs were found (*cysC*, *purC*, *purM* and *purN*) while at 1000 m it was only *purE*.

Other categories

The percentage of "biosynthesis of other secondary metabolites" AMGs was significantly differed at 1000 m than 50 m and 75 m stations (one-way ANOVA, $p<0.05$), being higher at 1000 m (post hoc Tukey tests). The percentage reached 10 and 14% at LV10 and LV18, respectively, while it was 7±2% considering all other depths (Table 4). The "staurosporine biosynthesis" category was particularly important at all stations, involving genes *prnA*, *rebH* and *ktzQ* (Supplementary Table 2). It is noteworthy that only at LV10 at 1000 m the category of "prodigiosin biosynthesis" was detected (17% of all "biosynthesis of other secondary metabolites" AMGs, Supplementary Table 2) involving the gene *fabG*.

AMGs involved in "folding, sorting and degradation" (all cases; "sulfur relay system" and particularly the gene *mec*) significantly differed between 1000 m and 50 m stations (one-way ANOVA, $p<0.05$). Higher contribution was seen at 1000 m than 50 m (post hoc Tukey tests (Table 4).

The category "metabolism of other amino acids" included mainly "glutathione metabolism" AMGs (*PDG*, *gnd*, *gntZ*, *G6PD*, *zwf*), showed irregular levels and did not differ between the sampling depths (Table 4).

AMGs involved in "metabolism of terpenoids and polyketides" contributed at all cases <6% of all AMGs (Table 4) and consisted of genes related to "polyketide sugar unit biosynthesis" and "biosynthesis of vancomycin group antibiotics". Similarly to "nucleotide metabolism", exception was the station LV10 at 75 m where "geraniol degradation" AMGs was found as well.

AMGs associated to "xenobiotics biodegradation and metabolism" were minor, except for three surface samples; LV10 at 5 m and LV13 at 5 and 50 m (Table 4). Genes here involved "benzoate degradation" (*gcdH*, *pcaC* and *mhpE*). AMGs associated to "other amino acids metabolism" were minor and consisted mainly of "glutathione metabolism" genes.

2 Supplementary Figures and Tables

Supplementary Table 1. Output of permutational analysis of variance (PERMANOVA) testing variation among sampling stations. Salinity (*S*), temperature (T), density (D), and the concentrations of phosphate (PO_4^{3-}), the sum of nitrate and nitrite (NOx), silicate (SiO_4), total nitrogen (TN) and phosphorus (TP), dissolved oxygen (DO), dissolved organic carbon (DOC), phosphorus (DOP) and nitrogen (DON) and chlorophyll a (Chl) at the sampling stations.

**Source of variation**	**df**	**MS**	**Pseudo-F**	**p**
Physicochemical variables: NOx, PO_4^{3-}, SiO_4, DO, DOC, DOP, DON, TN, TP				

Station	3	11.58	0.95	0.34
Residuals	10	12.13		
Physicochemical and biological variables: NOx, PO_4^{3-}, SiO_4, DO, DOC, DOP, DON, TN, TP and Chl and BP				
Station	3	14.02	1.00	0.30
Residuals	10	13.99		
Abundances: virus-like particles, heterotrophic and autotrophic bacteria				
Station	3	41.26	0.40	0.91
Residuals	10	102.13		
Viral community: family level				
Station	3	135.07	1.50	0.23
Residuals	10	89.96		
Viral community: genus level				
Station	3	187.25	1.24	0.23
Residuals	10	150.38		
Potential bacterial hosts of viruses				
Station	3	146.64	1.28	0.26
Residuals	10	114.55		
AMG major metabolic pathways				
Station	3	1376.2	0.89	0.52
Residuals	10	1552.9		

Supplementary Table 2. a. Alpha-diversity indices (richness, Chao1, ACE, Shannon's H, Simpsons, inverse Simpsons, Fisher, and Pielou's J) for the sampling stations, as calculated by MetaPop pipeline. **b.** Number of reads of some rare viral genera in all sampling stations.

a.

**Station**	**Depth**	Observed	Chao1	se.Chao1	ACE	se.ACE	Shannons_H	Simpson	InvSimpson	Fisher	Peilous_J
LV3	5	50689	52036.31	63.83	51689.98	107.99	10.39	1.00	19233.08	13285.19	0.96
LV3	50	52039	52796.40	43.65	52646.25	107.91	10.41	1.00	19156.50	13662.51	0.96
LV3	75	51914	53118.45	54.94	53122.12	97.29	10.48	1.00	22950.37	15499.96	0.96
LV10	5	50120	51551.42	65.97	51219.39	107.25	10.38	1.00	18882.53	13243.62	0.95
LV10	50	51892	52673.73	45.10	52470.92	107.29	10.43	1.00	20081.29	13750.77	0.96
LV10	75	50916	52862.81	77.08	52624.16	102.65	10.41	1.00	20518.98	14458.87	0.96
LV10	1000	40024	52935.05	283.25	54360.30	120.64	9.99	1.00	8126.28	14799.67	0.92
LV13	5	51617	52473.05	47.13	52318.05	108.97	10.44	1.00	20867.24	13561.51	0.96
LV13	50	51203	52628.92	60.28	52704.42	93.49	10.44	1.00	20078.01	15679.49	0.96
LV13	75	52166	52794.98	38.71	52684.27	106.43	10.48	1.00	22121.80	14178.76	0.96
LV18	5	49710	52777.66	97.30	53000.87	88.41	10.43	1.00	19971.70	16968.91	0.96
LV18	50	50643	53583.28	95.73	53614.88	91.26	10.42	1.00	17275.93	16625.99	0.96
LV18	75	40677	49204.71	209.58	49563.84	102.63	10.14	1.00	10621.03	14538.17	0.94

LV18	1000	4376	5512.45	98.88	5513.49	37.36	7.74	1.00	1272.88	933.68	0.85

b.

Station	Depth	Bcep22	Bpp1	Cba41	Cp220	Chloro	Kp36	Lambda	Luz24	M12	Mu	Nit1	P12002	P12024	P70	Prtb	Prymnesio	Septima	Slash	T4	Vegas	Vp5
LV3	5	283	138	151	48	39	48	2671	82	909	17	134	150	83	24	20	14	223	12	2131	4	293
LV3	50	438	274	252	66	57	81	4455	99	1312	17	243	273	214	42	30	22	371	27	2754	5	496
LV3	75	230	166	168	31	33	53	2586	82	699		127	180	98	41	27	14	232	12	2081	6	377
LV10	5	432	287	255	112	80	68	3951	110	1555	22	216	284	180	41	37	26	347	29	3670	7	403
LV10	50	345	284	208	66	36	68	3935	101	991	34	178	278	165	37	36	27	329	13	2241	4	460
LV10	75	294	270	327	60	53	111	3371	110	757	18	158	291	164	127	47	16	270	26	2740	5	495
LV10	1000	131	23	94	17	34	46	1377	99	330	46	81	54	62	22	43	12	152				
LV13	5	374	249	282	130	102	54	3836	128	1544	13	211	272	206	129	40	35	317				
LV13	50	314	228	237	61	59	71	3411	117	1025	45	201	226	184	115	45	16	773	23	180		
LV13	75	234	195	166	30	36	57	2656	96	665	25	113	192	102	84	27	121	4167	8	434		
LV18	5	183	177	158	49	40	28	2484	84	579	68	110	179	90	61	23	57	2515	7	420		283
LV18	50	202	195	221	52	40	21	2667	80	560	68	95	185	82	57	36	37	1804	3	308	2	307
LV18	75	158	192	208	15	39	59	2200	97	394	32	121	174	87	59	48	12	207	24	1377	6	307
LV18	1000	71	9	124	28	34	70	725	105	207	36	64	21	68	21	43	9	72	11	589	27	84

Supplementary Table 3. Percentage contribution of the specific KEGG metabolic pathways over the total AMG reads identified at the sampling stations. Presented AMG categories are involved in following tables **A:** amino acid metabolism, **B:** carbohydrate metabolism, **C:** cofactors and vitamins metabolism, **D:** energy metabolism, **E:** lipid metabolism, **F:** glycan biosynthesis and metabolism, **G:** nucleotide metabolism, and **H:** other rarer pathways. The first two columns in the following table A (red font) are the head-columns for all remaining tables.

A: amino acid metabolism

**Station**	**Depth**	**Alanine, aspartate and glutamate metabolism**	**Glycine, serine and threonine metabolism**	**Cysteine and methionine metabolism**	**Lysine biosynthesis**	**Valine, leucine and isoleucine degradation**	**Arginine and proline metabolism**	**Phenylalanine metabolism**
LV3	5	7	5	52	0	0	35	2
LV3	50	4	3	56	0	0	37	0
LV3	75	3	0	67	0	0	29	0
LV10	5	7	9	45	0	0	38	0
LV10	50	6	0	55	0	0	39	0
LV10	75	3	0	64	0	1	32	0
LV10	1000	5	5	70	0	0	20	0
LV13	5	9	10	49	1	0	30	2
LV13	50	3	1	68	0	0	28	0
LV13	75	5	2	57	0	0	34	2
LV18	5	5	0	53	0	0	40	2
LV18	50	5	0	56	0	0	38	2
LV18	75	11	0	59	0	0	30	0
LV18	1000	15	0	69	0	0	15	0

B: carbohydrate metabolism

Glycolysis / Gluconeogenesis	Pentose phosphate pathway	Pentose and glucuronate interconversions	Fructose and mannose metabolism	Galactose metabolism	Ascorbate and aldarate metabolism	Starch and sucrose metabolism	Amino sugar and nucleotide sugar metabolism	Pyruvate metabolism	C5-Branched dibasic acid metabolism	Butanoate metabolism	Glyoxylate and dicarboxylate metabolism
0	3	8	13	18	8	0	46	0	0	0	5
0	4	4	15	21	4	0	49	0	0	0	2
4	8	13	4	17	13	4	38	0	0	0	0
0	2	5	19	19	5	0	49	0	0	0	1
0	3	10	10	17	10	0	50	0	0	0	0
0	2	13	15	9	13	0	46	0	0	2	0
0	0	8	25	0	8	0	42	17	0	0	0
0	8	5	14	21	5	0	44	0	0	0	3
2	16	4	14	14	4	2	41	0	2	0	0
0	25	6	16	6	6	3	34	0	0	0	3
0	12	12	18	12	6	0	41	0	0	0	0
0	21	5	0	26	5	0	42	0	0	0	0
0	0	0	23	15	0	0	62	0	0	0	0
0	0	0	27	9	0	0	64	0	0	0	0

C: cofactors and vitamins metabolism

Thiamine metabolism	Riboflavin metabolism	Nicotinate and nicotinamide metabolism	Biotin metabolism	Folate biosynthesis	One carbon pool by folate	Porphyrin and chlorophyll metabolism	Ubiquinone and other terpenoid-quinone biosynthesis
0	13	0	0	19	16	53	0
0	9	5	0	23	14	47	2
0	11	11	0	6	17	56	0
0	7	4	0	37	7	43	3
0	18	7	0	14	7	50	4
0	10	6	0	13	39	26	6
0	0	0	67	0	0	33	0
1	5	3	1	36	9	41	3
0	8	15	3	15	8	49	3
0	10	0	0	15	20	55	0
0	11	6	0	44	11	28	0
0	8	16	0	36	8	28	4
0	8	15	0	23	23	23	8
0	0	0	20	40	0	40	0

D: energy metabolism

Oxidative phosphorylation	Photosynthesis	Carbon fixation in photosynthetic organisms	Methane metabolism	Sulfur metabolism
0	73	0	9	18
8	72	0	4	16
0	95	0	0	5
0	64	3	11	22
4	74	0	0	22
0	90	0	0	10
0	0	0	67	33
2	56	4	15	23
0	77	3	0	21
0	90	5	5	0
0	88	0	0	13
0	89	0	0	11
0	100	0	0	0
0	0	0	0	0

E: lipid metabolism

Fatty acid biosynthesis	Biosynthesis of unsaturated fatty acids
0	100
0	0
0	0
0	100
0	100
0	100
100	0
33	67
100	0
0	0
0	0
0	0
0	0
100	0

F: glycan biosynthesis and metabolism

N-Glycan biosynthesis	Various types of N-glycan biosynthesis	Glycosphingolipid biosynthesis - lacto and neolacto series	Lipopolysaccharide biosynthesis
0	0	0	100
0	0	0	100
20	0	0	80
0	0	0	100
33	0	0	67
20	0	0	80
17	17	0	67
11	0	5	84
11	0	11	78
17	0	0	83
17	0	0	83
0	0	0	100
0	0	0	0
33	0	0	67

G: nucleotide metabolism

Purine metabolism	Pyrimidine metabolism
100	0,0
100	0,0
100	0,0
100	0,0
100	0,0
96	4,2
100	0,0
100	0,0
100	0,0
100	0,0
100	0,0
100	0,0
100	0,0
100	0,0

H: other AMGs

Thiamine metabolism	Riboflavin metabolism	Nicotinate and nicotinamide metabolism	Biotin metabolism	Folate biosynthesis	One carbon pool by folate	Porphyrin and chlorophyll metabolism	Ubiquinone and other terpenoid-quinone biosynthesis
0	13	0	0	19	16	53	0
0	9	5	0	23	14	47	2
0	11	11	0	6	17	56	0
0	7	4	0	37	7	43	3
0	18	7	0	14	7	50	4
0	10	6	0	13	39	26	6
0	0	0	67	0	0	33	0
1	5	3	1	36	9	41	3
0	8	15	3	15	8	49	3
0	10	0	0	15	20	55	0
0	11	6	0	44	11	28	0
0	8	16	0	36	8	28	4
0	8	15	0	23	23	23	8
0	0	0	20	40	0	40	0

H: other AMGs (continue)

Geranio degradation	Polyketide sugar unit biosynthesis	Biosynthesis of vancomycin group antibiotics
0	67	33
0	60	40
0	60	40
0	64	36
0	60	40
50	50	0
0	50	50
0	62	38
0	67	33
0	63	38
0	60	40
0	100	0
0	0	0
0	50	50

H: other AMGs (continue)

Streptomycin biosynthesis	Acarbose and validamycin biosynthesis	Staurosporine biosynthesis	Prodigiosin biosynthesis	Biosynthesis of secondary metabolites - other antibiotics	Nitrotoluene degradation	Sulfur relay system
32	26	37	0	5	0	0
38	33	24	0	5	0	0
36	18	36	0	9	0	0
37	27	33	0	3	0	0
30	25	35	0	10	0	0
14	0	86	0	0	0	0
17	17	50	17	0	0	0
36	30	30	0	5	0	0
39	22	33	0	6	0	0
31	31	31	0	8	0	0
20	20	50	0	10	0	0
0	0	100	0	0	0	0
0	0	100	0	0	0	0
14	14	71	0	0	0	0

H: other AMGs (continue)

Cyanoamino acid metabolism	beta-Alanine metabolism	Glutathione metabolism
67	0	33
50	0	50
0	33	67
50	0	50
0	0	100
0	0	100
0	0	0
30	0	70
0	0	100
14	0	86
0	0	100
0	0	100
0	0	0
0	0	0

Supplementary Figures

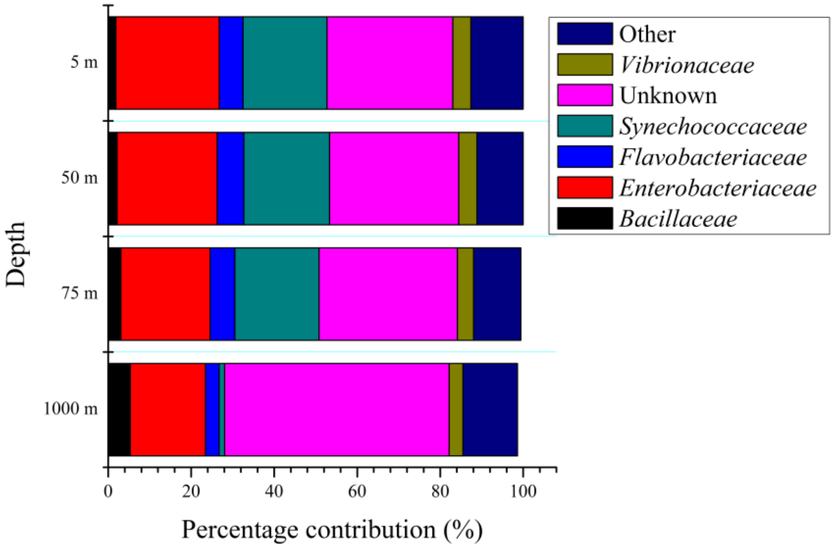

Supplementary Figure 1. Percentage contribution of the major potential host bacterial families in the sampling stations. “Other” families contribute <0.1% to the total reads. Average values derive from the four sampling stations (LV3, LV10, LV13 and LV18).
